# The Outcome of Agitation in Poisoned Patients in an Iranian Tertiary Care University Hospital

**DOI:** 10.1155/2014/275064

**Published:** 2014-12-04

**Authors:** Ali Mohammad Sabzghabaee, Ahmad Yaraghi, Elham Khalilidehkordi, Seyyed Mohammad Mahdy Mirhosseini, Elham Beheshtian, Nastaran Eizadi-Mood

**Affiliations:** ^1^Isfahan Clinical Toxicology Research Center, Isfahan University of Medical Sciences, Isfahan, Iran; ^2^Department of Anesthesiology and Critical Care, School of Medicine, Isfahan University of Medical Sciences, Isfahan, Iran; ^3^Department of Clinical Toxicology, Noor and Ali-Asghar (PBUH) University Hospital, Isfahan University of Medical Sciences, Isfahan, Iran

## Abstract

*Introduction*. This study was conducted to evaluate and document the frequency and causes of agitation, the symptoms accompanying this condition in intoxications, relationship between agitation score on admission and different variables, and the outcome of therapy in a tertiary care referral poisoning center in Iran. *Methods*. In this prospective observational study which was done in 2012, 3010 patients were screened for agitation at the time of admission using the Richmond Agitation Sedation Scale. Demographic data including age, gender, and the drug ingested were also recorded. The patients' outcome was categorized as recovery without complications, recovery with complications (hyperthermia, renal failure, and other causes), and death. *Results*. Agitation was observed in 56 patients (males, *n* = 41), mostly aged 19–40 years (*n* = 38) and more frequently in illegal substance (stimulants, opioids and also alcohol) abusers. Agitation score was not significantly related to the age, gender, and previous history of psychiatric disorders. Forty nine patients had recovery without any complication. The need for mechanical ventilation was the most frequent complication. None of the patients died. *Conclusion*. Drug abuse seems to be a must-to-consider etiology for patients presenting with acute agitation and its morbidity and mortality could be low in agitated poisoning cases if prompt supportive care is performed.

## 1. Introduction

Agitation is defined as restlessness accompanied by excessive and aimless motor or cognitive activity, which is usually experienced by tension and anxiety [1, 2]. The underlying causes of agitation fall into five most prevalent categories including neurological diseases, drug intoxication or withdrawal symptoms, psychological disorders, metabolic diseases, and infections [3, 4]. Clinically, in intoxicated cases agitation is presented as frequent movements of head and limbs and attempt for extubation despite attempts of the staff to calm the patient [5]. Agitation could lead to different complications such as malignant hyperthermia, rhabdomyolysis, renal failure, and even death [6].

Several agents and conditions are suspected to cause agitation after poisoning or overdose [7, 8]. These include the abuse of drugs such as cocaine, amphetamine, and hallucinogens; the withdrawal syndrome of alcohol, sedatives, and opioids; and intoxication with centrally acting anticholinergic drugs, antihistamines, tricyclic antidepressants, neuroleptics, monoamino oxidase inhibitors, and salicylic acid. Previous studies on patients with antihistamine and methamphetamine intoxication have shown that only some of these patients experience agitation [9, 10]. Moreover, agitation has been reported as an unusual presentation in intoxication of some drugs such as baclofen, olanzapine, phenytoin, risperidone, aripiprazole, adrenaline, and abuse of dextromethorphan [11–17].

Studies on agitated critically ill patients hospitalized in intensive care units (ICUs) have shown that presence of agitation is associated with prolonged duration of hospitalization in ICU, higher prevalence of nosocomial infections, unplanned extubation, higher morbidity rate, and higher hospital costs [3, 4, 18]. Jaber and colleagues have previously found that the abuse of sedative drugs, neglected hyperthermia, untreated hyponatremia or hypernatremia, alcohol abuse, and the underlying psychiatric disorders are all risk factors for agitation.In their study, patients' age was not found to be a risk factor for this clinical condition [4]. Sever agitation may also cause rhabdomyolysis, renal failure, and hyperthermia [1].

Considering the importance of the outcome of agitation for the treatment in poisoned patients and the necessity of its early diagnosis to reduce the morbidity and mortality caused by this condition, current study was conducted to evaluate and document the frequency and causes of agitation, the symptoms accompanying this condition in intoxications, relationship between agitation score on admission and different variables, and the outcome of therapy in a tertiary care referral poisoning center in Iran.

## 2. Material and Methods

In this prospective observational study, which was done in the department of toxicological emergencies of Noor and Ali-Asghar (PBUH) University hospital during the year 2009, all poisoned patients either intentionally or accidentally who were referred to the poisoning emergency department and had clinical signs of agitation [19] at the time of admission were included. The study protocol was approved by the institutional board of human studies at Isfahan University of Medical Sciences. In addition, after the study design was accurately explained to each patient, informed consent was taken from them for inclusion to this study. If the patient was not able or had not the capacity for decision making, informed consent for inclusion to this study was taken from the patients' first degree family.

We used a nonprobability method for sampling and patients were enrolled consecutively. Patients who were discharged on their personal will and those who had agitation due to reasons other than acute intoxication (based on a documented neurology consultation and brain computer tomography scan) were excluded from the study.

After performing primary clinical evaluations and providing basic supportive care for the patients, the agitation score was determined by a medical toxicologist using the Richmond agitation sedation scale (RASS) in patients who met the inclusion criteria. RASS for the patients was evaluated within 30–60 seconds using three phases of observation, response to verbal simulation, and response to physical simulation. RASS scale is a 10-point scale ranging from combative (+4) to unarousable (−5). The scores +1 to +4 are used to estimate agitation and anxiety, 0 denotes the calm and alert state, and −1 to −5 denote sedation [20]. Demographic data for all patients including age, gender, and the drug ingested were also recorded.

After the documentation of data and RASS score all patients underwent routine treatment protocol for agitation control according to our institutional guidelines which was intravenous midazolam (0.1–0.3 mg/kg) and restraint of the agitated patient. This bolus dose was repeated or followed by infusion of midazolam (0.05 mg/kg–0.3 mg/kg). If the patients did not respond to this therapeutic measure, an intravenous dose of sodium thiopental (1-2 mg/kg) followed by a 1 mg/kg infusion was started. The airway, respiration, and blood pressure were also checked closely and patients were transfer to the poisoning ICU for closer monitoring if needed.

The patients' outcome was categorized as recovery without complications, recovery with complications (hyperthermia, renal failure, and other causes), and death. Collected data were analyzed using SPSS software version 13.0 (SPSS Inc., Chicago, IL, USA). Means were compared using independent Student's *t*-test. The probable statistical relationship between the agitation score and the outcome was determined using the Spearman's correlation test. The median values for the ordinal variables were compared using the Mann-Whitney *U* test, and the frequency distribution of agitation with regard to different factors was compared using the Chi-square test. *P* value less than 0.05 was considered significant.

## 3. Results

Among 3010 poisoning patients admitted to our poisoning referral center during the study period, agitation was observed in 56 patients at the time of admission. The highest prevalence of agitation was observed in the age group of 19–40, consisting of 67.9% of all the cases. There was not statistical deference for the presence of agitation in patients with positive past medical history for psychiatric disorders (*n* = 12) and patients without it (*n* = 40) or with unknown history (*n* = 4) (*P* = 0.24).

Agitation was more common in men (73.2%). Comparison of the median value of agitation score on admission indicated that the groups were not significantly different in this respect (*P* = 0.114).

The mean agitation scores in patients with positive and negative history of psychiatric disorders were 1.9 ± 0.90 and 2.3 ± 0.93 (*P* value <0.05), respectively. The median agitation score was 2 for the both groups (*P* = 0.245).

Agitation was observed in 33.4% of the patients following illegal substance abuse (stimulants, alcohol, and opioids) (Table 1). The highest mean agitation score obtained was 3, which was observed in opioid intoxications (tramadol intoxication and those patients received naloxone after opioid intoxication). The results regarding the clinical symptoms and paraclinical evaluation have been shown in Tables 2 and 3. Agitation score was not significantly related to the age, gender, and previous history of psychiatric disorders (*P* > 0.05). Length of hospital stay was between 2 and 24 hours. Forty nine patients had recovery without any complication. The need for mechanical ventilation was the most frequent complication in our agitated patients (Table 4).

## 4. Discussion

This study was performed to evaluate the causes and outcome of agitation in poisoning patients and determine the relationship between agitation score on admission and different variables.

Our results showed that the highest prevalence of intoxicated patients with agitation was in the age range of 19–40 which is not consistent with a previous study that reported this in a lower range of age [21]. According to our personal experience after doing many discharge interviews with these patients we think that this high prevalence of intoxication with agitation in young adults may be attributable to the identity issues, the gap between children's values and their parents', the high economical inflation rate, and unemployment. In a study performed in an eighteen-bed MICU of a tertiary care center, it was also found that the age was not a risk factor for occurrence of agitation [22]. It should be mentioned that most of the patients referred to our center were male and the underlying causes for most cases of agitation were opioids cases receiving naloxone which could be justified by the higher prevalence of opioid addiction in men [23, 24].

Although agitation has not been reported in opioid intoxications, the addicts may experience agitation in case of receiving excessive doses of naloxone. In the current study, seven patients received naloxone before being referred by the emergency ambulance services and three patients were agitated following intake of oral doses of naltrexone. Also tramadol intoxication may cause agitation in some patients.

Anticonvulsants, antipsychotics and TCAs with their anticholinergic effects, amphetamines with their sympathomimetic effects, diphenoxylate (opioid) with its atropine ingredient, pesticides, and antihypertensions can cause agitation as is shown in this study and also by others [25–28].

Most of the patients had normal vital signs on admission and their agitation score was less than 2 (62.5%). Few patients had tachycardia as expected in patients with agitation. Low median score of agitation may be due to the small amount of ingested dose of drug. In our study, some patients had some levels of decreased consciousness that all of them recovered without complications and it can be justified with agitating score less than 2 observed in our patients.

The complications were seen in 7 patients of whom 3 had agitation score more than 2. No mortality was observed. Low morbidity and mortality rate might be due to low toxicity level as presented by low number of agitations score in most of the patients.

In our study we did not evaluate the severity of poisoning according to perceived stress scale (PSS) scoring system which could be considered a limitation. We recommend further with a larger sample size for determining the cut-off point score of agitation in predicting tachycardia and complications.

## 5. Conclusions

Drug abuse seems to be a must-to-consider etiology for patients presenting with acute agitation and its morbidity and mortality could be low in agitated poisoning cases if prompt supportive care is performed.

## Figures and Tables

**Table 1:** Frequency distribution of agitation with respect to the ingested toxin in studied patients.

Drug	*N* (%)
Illegal substance abuse	19 (33.4)
Multidrug abuse^*^	9 (16.2)
Benzodiazepines	9 (16.2)
Anticonvulsants	7 (12.6)
Tricyclic antidepressants	6 (10.8)
Antipsychotics	3 (5.4)
Antihypertensives	2 (3.6)
Pesticides	1 (1.8)

Total	56 (100)

^*^Multidrug abuse: abusive ingestion of more than one medication.

**Table 2:** Frequency distribution of clinical symptoms of poisoned patients presenting with agitation.

Variables	Range	Number (%)
Vital signs		
Heart rate (per minute)	60–100	47 (89.3)
	<60	4 (7.1)
	>100	2 (3.6)
Respiratory rate (per minute)	12–20	47 (83.93)
	<12	4 (7.14)
	>20	5 (8.93)
Systolic blood pressure (mmHg)	90–140	47 (83.93)
	<90	4 (7.14)
	>140	5 (8.93)
Body temperature (°)	35–38°C	56 (100)

Pupils' size	3–5 mm	40 (71.4)
	<3 mm	9 (16.1)
	>5 mm	7 (12.5)

Consciousness level	Conscious	21 (37.5)
	Lethargic	28 (50)
	Obtundation	4 (7.1)
	Stupor or coma	3 (5.4)

**Table 3:** Frequency distribution of paraclinical tests in the studied patients.

Parameter	Range	*N* (%)
Blood urea nitrogen (BUN); normal range: 6–20 (mg/dL)	>20	2 (3.6)
Serum creatinine (Cr) level; normal range: 0.6–1.3 (mg/dL)	>1.3	4 (7.1)
Serum Na^+^ level; normal range: 135–145 (mmol/L)	<135	5 (8.9)
	>145	2 (3.6)
Serum K^+^ level; normal range: 3.5–5 (mmol/L)	<3.5	5 (8.9)
	>5	2 (3.6)
Blood glucose level; normal range: 70–110 (mmol/L)	>110	2 (3.6)
	<70	18 (32.1)
Serum pH value; normal range: 7.35–7.45	>7.45	1 (1.8)
Blood oxygen saturation level; normal range (more than 90%)	<90%	2 (3.6)
Hemoglobin level; normal range: 12–14 g/dL (women) 14–16 g/dL (men)	>14 or >16	13 (23.2)
White blood cell count (WBC); normal range: 4.5–11 (×10^3^/*µ*L)	<4.5	4 (7.1)
	>11	6 (10.7)
Platelet count; normal range: 150–450 (×10^9^/L)	<150	11 (19.6)

**Table 4:** Outcomes of therapy for study patients admitted with agitation regarding the agitation score.

		Agitation score				Total
		1.00	2.00	3.00	4.00	1.00
Recovery without complications		14 (28.6)	17 (34.7)	14 (28.6)	4 (8.2)	49 (100)

Recovery with complications	Hyperthermia	0 (0)	1 (100)	0 (0)	0 (0)	1 (100)
	Renal failure	0	0	1 (100)	0	1 (100)
	Aspiration Pneumonia	0 (0)	1 (100)	0 (0)	0 (0)	1 (100)
	Intubation and ventilation	1 (25)	1 (25)	1 (25)	1 (25)	4 (100)

Total		15 (26.8)	20 (35.7)	16 (28.6)	5 (8.9)	56 (100)

The results are presented as number of patients (%).
